# Supplementary material for: Preoperative Clinical Phenotyping for Individualised Rehabilitation in End-Stage Knee Osteoarthritis
Source: J Funct Morphol Kinesiol. 2025 Sep 19;10(3):360. doi: 10.3390/jfmk10030360 (PMC12452372; doi:10.3390/jfmk10030360)
Supplement: Supplementary file 1 [file jfmk-10-00360-s001.zip › Supplimentsary File S1.pdf]

Extracted from the Human Research Ethics Committee approved study protocol:

Sampling technique

A stratified random sampling technique will be used for this study. This will provide a sample that is representative of the subpopulation groups found amongst the individuals awaiting knee arthroplasty and will improve the accuracy in estimation of the outcomes (e.g. female vs male) [1,2]. The proportion of the sample which will be taken from each of the final strata will be determined by proportion allocation, meaning each strata will be sampled based on their respective representation in the population to ensure that the final sample resembles the composition of the population (Frey, 2018).

The following diagram illustrates the proposed sampling strata (based on the sampling pool):

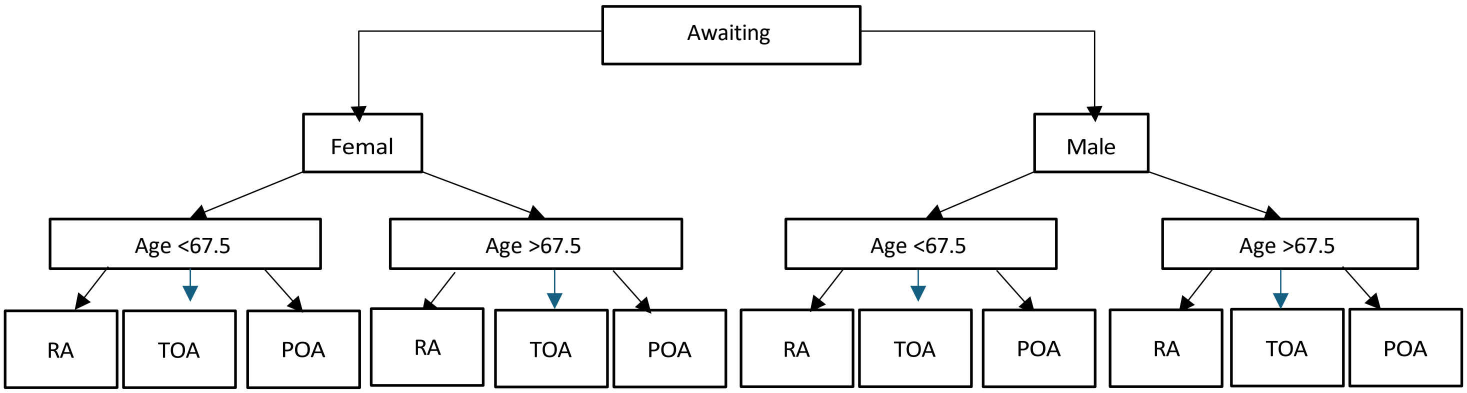

*Age categories according to median age; RA rheumatoid arthritis; TOA traumatic OA; POA primary OA

Figure: Stratification for sample

Strata identification

The individuals who were included in the desk review from the first phase of this project (and therefore have been subjected to the initial inclusion/ exclusion criteria) will be used for sampling in this study:

- Firstly, this group of individuals will be divided into male and female strata due to the global prevalence of knee OA being higher in females than in males [3].
- Next, the male and female strata will be divided into age related strata due to expected differences in functional priorities based on life roles (e.g. working individual vs grandparents). In addition, the prevalence of OA typically increases with age and some studies have linked age with worse clinical and structural trajectories [4].
- Lastly, the categories based on rheumatoid OA, traumatic OA, primary OA will be divided

A simple random sample will be taken from the final strata according to the percentage of representation in the entire group.

Data collection setting

Data will be collected at the FNB 3-D Motion Analysis Laboratory at Stellenbosch University Tygerberg Health Sciences Campus in Cape Town.

Recruitment method

Individuals who has given consent to be contacted for research purposes in phase one of this project and has been randomly selected from the stratification model will be contacted via telephone, explaining the purpose of the phone call and the study, and invited to take part in this study. Individuals will be offered time to think about their willingness to participate if they are unsure and a follow-up phone call will be made with their permission. If they verbally consent to take part, a suitable date will be diarised for assessment at the FNB 3-D Motion Analysis Laboratory at Tygerberg Campus, Stellenbosch University. Participants will be reminded of their appointment per phone call or SMS (as per their preference).

Data to be collected and tools

Based on systematic reviews and feasibility studies on structural/ functional disease progression and patient subgroups in OA [4-6], the following clinical, functional, biomechanical outcomes and self-reported outcome measures has been chosen. The clinical, functional and self-reported measures were chosen for their ability to assess components related to the various clinical subgroups from international literature. In addition, literature on supported self-management for OA and other chronic conditions were consulted for measures related to important components of self-management such as coping behaviour and self-efficacy [7-9]

VICON procedures

The VICON 3D Motion Analysis (© Vicon Motion Systems Ltd UK) system will be used for capturing the gait movement. The T-series camera system has high speed accuracy and a resolution of 1-maga pixels (1120 x 896) capturing 10-bit grey scale images at 250 frames per second and does marker tracking of up to 800Hz. Retro-reflective markers will be applied to key areas to capture the kinematic and kinetic information. The researcher will be trained in marker placement by the Central Analytics Facility (CAF) at the University of Stellenbosch and all marker placements will be done by the researcher herself for every study participant to reduce the risk of bias. The VICON T-series setup at the FNB 3-D Motion Analysis Laboratory at Stellenbosch

University Tygerberg Health Sciences Campus in Bellville consists of a 10 meter walkway amongst 8 infrared cameras and a force plate for capturing ground reaction force in the center of the walkway. Each end of the walkway (marked with a line on the floor) there is a 1 m space which can be utilized for turning around.

Preparation:

Participants will be requested to wear shorts and expose their lower abdomen (ASIS) for marker placement. All the areas of the skin where the markers will be placed will be cleaned with alcohol in order for the markers to stay on the skin during the gait analysis. The retro-reflective markers of 9.5 mm diameter will be placed at various anatomical landmarks on participants according to the Plug in Gait marker set (see below) using double sided tape.

Walking trail capture:

A minimum of six walking trails, barefoot and at a self-selected speed with/without their assistive devices will be completed. The participant will be instructed to walk as they would normally, ensuring that they cover the section with the force plate and continue to walk each round, making a turn at the end of the walkway. They are allowed to pause for a rest after the marked line and will be provided a chair to sit on if required. Al trail will be considered successful if the participant has placed one full length of a foot on the force plate, with no other foot contact at the same time and completed the trail. This will have to occur three times for the left foot and three times with the right foot.

After assessment:

The markers will be carefully removed from the participant's skin and they will be allowed to rest and take a break with refreshments.

Maker Label	Definition	Position	Type	Segment
(7	7th Cervical Vertebra	Spinous process of 7th cervical vertebra	**Anatomical**	**Torso**
**RBAK**	Right Back	Placed i|n the middle of the right scapula. Tliis marker has no symmetrical marker on the left side.	**Technical**	
**T1O**	10th Thoracic Vertebra	Spinous process of 10th thoracic vertebra	**Anatomical**	
**CLAV**	Clavicle	Jugular Notch where the clavicle meets the sternum	**Anatomical**	
**STRN**	Sternum	Xiphoid process of the Sternum	**Anatomical**	
**LASI**	Left Anterior S1uperior Iliac Spine	Placed directly over the left anterior superior iliac spine	**Anatomical**	**Pelvis**
**RASI**	Right Anterior Siuperior Iliac Spine	Placed directly over the right anterior superior iliac spine	**Anatomical**	
**LPSI**	Left Posterior Siuperior Iliac Spine	Placed directly over the left posterior superior iliac spine	**Anatomical**	
**RPSI**	Right Posterior Superior Iliac Spice	Placed directly over the right posterior superior iliac spine	**Anatomical**	
**SACR**	Sacrum	Placed on the skin mid-way between tne posterior superior iliac spines.	**Technical**	
**LTHI**	Left Thigh	Place the marker over the proxiimall laterall 1/3 surface of the thigh, on tne coronal plane oftne femur.	**Technical**	**Left Femur**
**LKNE**	Left Knee	Placed on the left lateral femoral epiicondyle, along an imaginary line that passes through the transepicondylar **axis**	**Anatomical**	
**LKAD**	Left Mediial Knee	Placed on the left mediial femoral epiconc!yle, along **an** imaginary line that passes through the transepicondylar axis	**Anatomical**	
**LSHI**	Left Shin	Placed on the flat, bony area below the knee on the medial surface of the tibial shaft.	Technical	Left Tibia
**LANK**	Left Ankle	Placed on the lateral malleolus along an imaginary line that passes thrnugh the transmalleol lar axis.	**Anatomical**	
**LMMA**	Left Mediial Malleolus	Placed on the styloid process of the medial malleolus along an imaginary line that passes.through the transmalleolar axis.	**Anatomical**	
**LHEE**	Left Heel	Placed on the calcaneus at the same height above tne plantar surface of the foot as the toe marker	**Anatomical**	Left Foot
**LTOE**	Left Toe	Placed over the second metatarsal head, on the mid-foot side of the equinus break between fore-foot and mid-foot.	**Anatomical**	

**RTHI**	Right Thigh	Place the marker over the proximal lateral 1/3 surface of the thigh, on the coronal plane of the femur.	**Technical**	**Right Femur**
**RKNE**	Right Knee	Placed on the left lateral femoral epicondyle, along an imaginary line that passes through the transepicondylar axis	**Anatomical**	
**RKAD**	Right Medial Knee	Placed on the left medial femoral epicondyle, along an imaginary line that passes through the transepicondylar axis	**Anatomical**	
**RSHI**	Right Shin	Placed on the flat, bony area below the knee on the medial surface of the right tibial shaft.	**Technical**	**Right Tibia**
**RANK**	Right Ankle	Placed on the lateral malleolus along an imaginary line that passes through the transmalleolar axis.	**Anatomical**	
**RMMA**	Right Medial Malleolus	Placed on the styloid process of the medial malleolus along an imaginary line that passes through the transmalleolar axis.	**Anatomical**	
**RHEE**	Right Heel	Placed on the calcaneus at the same height above the plantar surface of the foot as the toe marker	**Anatomical**	**Right Foot**
**RTOE**	Right Toe	Placed over the second metatarsal head, on the mid-foot side of the equinus break between fore-foot and mid-foot	**Anatomical**	

Outcome measures motivation:

i. Clinical outcome measures

A clinical examination of each individual will be performed and will include the following measures:

- **BMI calculation: Weight** (using a calibrated digital scale, individuals barefoot and wearing light clothing, measured to the nearest 0.5 kg) and **height** measurements (using a stadiometer to the nearest 0.1cm) [10,11]. Obesity has been linked to a metabolic subgroup of OA as well as worse clinical and structural progression trajectories and are used to assist in distinguishing clinically distinct phenotypes [4,12-14]. In addition, obesity is also an important psychosocial factor and has been considered a determinant in HRQol, functional outcome and mental health for people with knee OA [15-17].
- **Knee range of motion (ROM):** Passive knee **flexion** and **extension** ROM will be measured using a goniometer and a standard protocol (patient positioning and goniometer placement). One study found that lower knee flexion range (mean range 130 with SD of 12) were linked to worse clinical progression (related to pain and function) [13]. Although knee ROM is not typically linked to any specific clinical subgroups, it will be included for descriptive purposes of the population [4].
- **Muscle strength:** Knee **extension** and **flexion** muscle strength will be measured using a hand held dynamometer (HHD) following a standard protocol (patient position, trunk stabilisation and HHD placement). Three measurements will be taken and the mean measurement will be used. Muscle strength assists with distinguishing clinically distinct patients as it has been linked to subgroups related to biomechanical function as well as the presence of pain and worsening pain over time [4,5].

ii. *Functional outcome measures*

Functional tests will be used to determine the baseline functional level for individuals in this population and will be complimentary to our patient reported functional measures [18-20].

- **30 second Chair stand test (30-second CST):** Sit to stand/ stand to sit is a commonly impaired activity of daily living (ADL) for individuals with knee OA. The CST forms part of a recommended minimum core set of standardised performance-based tests for OA and it assesses lower body strength and dynamic balance during a change in body position [18,21]. In addition, this measure has also been used to reliably determine the falls risk in individuals with advanced knee OA [22,23]. This is a reliable and valid measure with a correlation co-efficient for test reliability of 0.93 - 0.97 in adults with advanced stage knee OA [21,24].

iii. *Biomechanical outcome measures*

A biomechanical gait analysis will be performed using the VICON 3D movement analysis system [25] with a standard plug-in gait model in order to describe the specific gait characteristics within the South African population of knee OA patients awaiting arthroplasty. The biomechanical features that are most commonly affected by OA has been reported in multiple studies [26-28] and has been chosen for evaluation and description in this population. The following information will be captured during a self-paced, barefoot gait:

- **Spatiotemporal parameters:** walking speed, stride length, stride duration, stance duration, base of support (BOS) and cadence will be captured and described for the population. In addition, the stride length and cadence will be evaluated according to cut off values identified by a previous study which found that these measures can accurately predict OA severity in clinical practice

- **Kinetic information:** Frontal and sagittal knee moments will be captured for use within the composite score as described below)
- **Kinematic information:** Frontal and sagittal knee angle information will be captured
- **Gait deviation index (GDI):** A composite gait score will be calculated using the GDI as this will reduce the multivariate gait information (nine frontal and sagittal knee moments and angles) into a single score for gait quality which can be used for comparison with normal gait and group differences within the population [29]. The GDI is typically used in populations with marked gait deviations and has been used in previous studies using populations with mild to moderate and moderate to severe radiographical knee OA [30,31]

iv. Patient reported outcome measures

It is important to capture the patient's perspective of their needs and limitations as the psychological and social aspects of living with chronic pain has a considerable impact on the success of management strategies within this population [16,32-34]. The following patient reported outcome measures will therefore be included:

- **Central Sensitisation Inventory (CSI):** Thirty percent of individuals with knee OA has pain sensitization which is indicative of central sensitization and represents a prominent subgroup of knee OA patients who requires a different treatment approach [4,5,35]. These individuals has a high risk of continued post-operative pain and worse clinical outcomes [36,37]. The CSI will be used for evaluation of the potential presence of pain sensitisation. The CSI has been used in previous studies for individuals with knee OA with moderate sensitivity and specificity. Individuals with pain sensitization requires a different approach to rehabilitation and are at risk for continued poor post-operative functional outcomes and this is a key concern and

are found amongst 30% of individuals with knee OA

- **The 10 item Center for Epidemiological Studies Depression score (Ces-D 10):** Depression has been linked to severe pain and worse clinical progression trajectories in patients with knee OA [12,14,38]. In addition, it has been associated with a chronic pain subgroup of OA patients who requires a different self-management and rehabilitation approach to address the chronic pain and mental health issues. This score has been validated for the detection of depression in South African among Afrikaans and Xhosa speaking individuals (internal consistency across samples (α = 0.69–0.89), Receiver Operator Characteristic curve good to excellent: 0.81 -0.94) [39]. The original Ces-D tool has been shown to be reliable in the detection of depression in individuals with knee OA and have been widely used in OA research [4,5].
- **The patient specific functional Score (PSFS):** This will be used to quantify activity limitation and functional outcomes as experienced by the participants as it will provide their view of their functional limitations in daily life. The PSFS has shown good reliability and high responsiveness in patients awaiting knee arthroplasty [40,41].
- **Oxford Knee Score (OKS):** The OKS has been developed for use in adults who will undergo TKA to assess knee specific function and pain [42]. It has also been used for monitoring disease progression in the knee OA population [43] It is a 12-item questionnaire with excellent test/ re-test reliability.
- **Brief COPE:** The use of passive coping strategies has been linked to worse clinical progression trajectories for individuals with knee OA [4]. Coping with a chronic pain condition is typically a key focus point of SSM interventions as it is aimed at improving passive coping behaviour and teaching active coping strategies [8]. The brief COPE is a 28-item questionnaire which assesses the active and passive coping strategies. Each item is scored on a

4-point Likert scale of frequency with which they are using these strategies. The outcome measure has adequate validity, re-test reliability and internal consistency.

- **Arthritis self-efficacy 8 item scale (ASES-8)**: Self-efficacy has been highlighted as a key focus area for SSM interventions as it is rooted in social learning theory aiming for behavioural change in the individual [8,44,45]. Self-efficacy is especially important for individuals with multi-morbidity [46]. The ASES-8 has been developed specifically for patients with arthritis and focusses on arthritis specific self-efficacy in managing pain, physical functional and other symptoms [44,47]. The ASES-8 has good reliability and validity in the knee OA population and will be used for determining self-efficacy of individuals in this study
- **Global physical activity questionnaire (GPAQ):** Physical activity levels have not yet been linked to worse clinical progression, but is however an indication of the lifestyle factors of an individual [14,48]. A 24% increase in risk for the development of cardiovascular disease have been identified in the knee OA population due to the reduced levels of activity as a result of chronic pain [49,50]. In addition, lower levels of activity may also be indicative of avoidance behaviour due to pain, which is seen as a passive coping strategy. Therefore, physical activity data will be collected using the GPA questionnaire which has good validity and reliability from previous knee OA as well as South African population studies.

References

1. Setia, M.S. Methodology series module 5: Sampling strategies. *Indian Journal of Dermatology* **2016**, *61*, 505-509, doi:10.4103/0019-5154.190118.
2. Frey, B.B. Stratified Random Sampling. **2018**, 1624-1624, doi:10.4135/9781506326139.
3. Conaghan, P.G.; Dickson, J.; Grant, R.L. Guidelines: Care and management of osteoarthritis in adults: Summary of NICE guidance. *BMJ* **2008**, *336*, 502-503, doi:10.1136/bmj.39490.608009.AD.
4. Deveza, L.A.; Melo, L.; Yamato, T.P.; Mills, K.; Ravi, V.; Hunter, D.J. Knee osteoarthritis phenotypes and their relevance for outcomes: a systematic review. *Osteoarthritis and Cartilage* **2017**, *25*, 1926-1941, doi:10.1016/j.joca.2017.08.009.
5. Dell'Isola, A.; Allan, R.; Smith, S.L.; Marreiros, S.S.P.; Steultjens, M. Identification of clinical phenotypes in knee osteoarthritis: a systematic review of the literature. *BMC Musculoskeletal Disorders* **2016**, *17*, 1-12, doi:10.1186/s12891-016-1286-2.
6. Dell'isola, A.; Wirth, W.; Steultjens, M.; Eckstein, F.; Culvenor, A.G. Knee extensor muscle weakness and radiographic knee osteoarthritis progression. *Acta Orthop* **2018**, *89*, 406-411, doi:10.1080/17453674.2018.1464314.
7. Aytekin, E.; Sukur, E.; Oz, N.; Telatar, A.; Eroglu Demir, S.; Sayiner Caglar, N.; Ozturkmen, Y.; Ozgonenel,

L. The effect of a 12 week prehabilitation program on pain and function for patients undergoing total knee arthroplasty: A prospective controlled study. *Journal of Clinical Orthopaedics and Trauma* **2019**, *10*, 345-349, doi:10.1016/j.jcot.2018.04.006.
8. Dineen-Griffin, S.; Garcia-Cardenas, V.; Williams, K.; Benrimoj, S.I. Helping patients help themselves: A systematic review of self-management support strategies in primary health care practice. *PLoS ONE* **2019**, *14*, 1-29, doi:10.1371/journal.pone.0220116.
9. Dube, L.; Rendall-Mkosi, K.; Van den Broucke, S.; Bergh, A.M.; Mafutha, N.G. Self-Management Support Needs of Patients with Chronic Diseases in a South African Township: A Qualitative Study. *Journal of Community Health Nursing* **2017**, *34*, 21-31, doi:10.1080/07370016.2017.1260983.
10. Cois, A.; Day, C. Obesity trends and risk factors in the South African adult population. *BMC Obesity* **2015**, *2*, 1-10, doi:10.1186/s40608-015-0072-2.
11. Peer, N.; Steyn, K.; Levitt, N. Differential obesity indices identify the metabolic syndrome in Black men and women in Cape Town: The CRIBSA study. *Journal of Public Health (United Kingdom)* **2016**, *38*, 175-182, doi:10.1093/pubmed/fdu115.
12. Collins, J.E.; Katz, J.N.; Dervan, E.E.; Losina, E. Trajectories and Risk Profiles of Pain in Persons with Radiographic, Symptomatic Knee Osteoarthritis: Data from the Osteoarthritis Initiative. *Osteoarthritis and Cartilage* **2014**, *22*, 622-630, doi:10.1038/jid.2014.371.
13. Holla, J.F.M.; Van Der Leeden, M.; Heymans, M.W.; Roorda, L.D.; Bierma-Zeinstra, S.M.A.; Boers, M.; Lems, W.F.; Steultjens, M.P.M.; Dekker, J. Three trajectories of activity limitations in early symptomatic knee osteoarthritis: A 5-year follow-up study. *Annals of the Rheumatic Diseases* **2014**, *73*, 1369-1375, doi:10.1136/annrheumdis-2012-202984.
14. Wesseling, J.; Bastick, A.N.; Ten Wolde, S.; Kloppenburg, M.; Lafeber, F.P.J.G.; Bierma-Zeinstra, S.M.A.; Bijlsma, J.W.J. Identifying trajectories of pain severity in early symptomatic knee osteoarthritis: A 5-year followup of the cohort hip and cohort knee (CHECK) study. *Journal of Rheumatology* **2015**, *42*, 1470-1477, doi:10.3899/jrheum.141036.
15. Kingsbury, S.R.; Corp, N.; Watt, F.E.; Felson, D.T.; O'Neill, T.W.; Holt, C.A.; Jones, R.K.; Conaghan, P.G.; Arden, N.K.; Adams, J.; et al. Harmonising data collection from osteoarthritis studies to enable stratification: Recommendations on core data collection from an Arthritis Research UK clinical studies group. *Rheumatology (United Kingdom)* **2016**, *55*, 1394-1402, doi:10.1093/rheumatology/kew201.
16. Lowry, V.; Ouellet, P.; Vendittoli, P.A.; Carlesso, L.C.; Wideman, T.H.; Desmeules, F. Determinants of pain, disability, health-related quality of life and physical performance in patients with knee osteoarthritis awaiting total joint arthroplasty. *Disability and Rehabilitation* **2018**, doi:10.1080/09638288.2017.1355412.
17. Vitaloni, M.; Botto-van Bemden, A.; Sciortino Contreras, R.M.; Scotton, D.; Bibas, M.; Quintero, M.; Monfort, J.; CarnÃ©, X.; de Abajo, F.; Oswald, E.; et al. Global management of patients with knee osteoarthritis begins with quality of life assessment: a systematic review. *BMC Musculoskeletal Disorders* **2019**, *20*, N.PAG-N.PAG, doi:10.1186/s12891-019-2895-3.
18. Dobson, F.; Bennell, K.L.; Hinman, R.S.; Abbott, J.H.; Roos, E.M.; Bennell, K. Recommended performance-based tests to assess physical function in people diagnosed with hip or knee osteoarthritis. *OARSI* **2013**.
19. Dobson, F.; Hinman, R.S.; Hall, M.; Marshall, C.J.; Sayer, T.; Anderson, C.; Newcomb, N.; Stratford, P.W.; Bennell, K.L. Reliability and measurement error of the Osteoarthritis Research Society International (OARSI) recommended performance-based tests of physical function in people with hip and knee osteoarthritis. *Osteoarthritis and Cartilage* **2017**, *25*, 1792-1796, doi:10.1016/j.joca.2017.06.006.
20. Mehta, S.P.; Morelli, N.; Prevatte, C.; White, D.; Oliashirazi, A. Validation of Physical Performance Tests in Individuals with Advanced Knee Osteoarthritis. *HSS Journal* **2019**, *15*, 261-268, doi:10.1007/s11420-019-09702-1.
21. Bennell, K.; Dobson, F.; Hinman, R. Measures of Physical Performance Assessments. *Arthritis Care and Research* **2011**, *63*, s350-370, doi:10.1002/acr.20538.
22. Roongbenjawan, N.; Siriphorn, A. Accuracy of modified 30-s chair-stand test for predicting falls in older adults. *Annals of Physical and Rehabilitation Medicine* **2020**, *63*, 309-315, doi:10.1016/j.rehab.2019.08.003.
23. Tsonga, T.; Michalopoulou, M.; Malliou, P.; Godolias, G.; Kapetanakis, S.; Gkasdaris, G.; Soucacos, P.

Analyzing the History of Falls in Patients with Severe Knee Osteoarthritis. *Clinics in orthopedic surgery* **2015**, *7*, 449-456, doi:10.4055/cios.2015.7.4.449.

24. Lin, Y.C.; Davey, R.C.; Cochrane, T. Tests for physical function of the elderly with knee and hip osteoarthritis. *Scandinavian Journal of Medicine and Science in Sports* **2001**, *11*, 280-286, doi:10.1034/j.1600-0838.2001.110505.x.
25. Ehara, Y.; Fujimoto, H.; Miyazaki, S.; Mochimaru, M.; Tanaka, S.; Yamamoto, S. Comparison of the performance of 3D camera systems II. *Gait & Posture* **1997**, *5*, 251-255, doi:https://doi.org/10.1016/S0966-6362(96)01093-4.
26. Biggs, P.R.; Whatling, G.M.; Wilson, C.; Metcalfe, A.J.; Holt, C.A. Which osteoarthritic gait features recover following total knee replacement surgery? *PLoS ONE* **2019**, *14*, 1-14, doi:10.1371/journal.pone.0203417.
27. Elbaz, A.; Mor, A.; Segal, G.; Debi, R.; Shazar, N.; Herman, A. Novel classification of knee osteoarthritis severity based on spatiotemporal gait analysis. *Osteoarthritis and Cartilage* **2014**, *22*, 457-463, doi:10.1016/j.joca.2013.12.015.
28. Mills, K.; Hunt, M.A.; Ferber, R. Biomechanical deviations during level walking associated with knee osteoarthritis: A systematic review and meta-analysis. *Arthritis Care and Research* **2013**, *65*, 1643-1665, doi:10.1002/acr.22015.
29. Schwartz, M.H.; Rozumalski, A. The gait deviation index: A new comprehensive index of gait pathology. *Gait and Posture* **2008**, *28*, 351-357, doi:10.1016/j.gaitpost.2008.05.001.
30. Kobsar, D.; Charlton, J.M.; Hunt, M.A. Individuals with knee osteoarthritis present increased gait pattern deviations as measured by a knee-specific gait deviation index. *Gait and Posture* **2019**, *72*, 82-88, doi:10.1016/j.gaitpost.2019.05.020.
31. Naili, J.E.; BrostrÃ¶m, E.W.; Clausen, B.; Holsgaard-Larsen, A. Measures of knee and gait function and radiographic severity of knee osteoarthritis - A cross-sectional study. *Gait & Posture* **2019**, *74*, 20-26, doi:10.1016/j.gaitpost.2019.08.003.
32. Moss, P.; Benson, H.A.E.; Will, R.; Wright, A. Patients with Knee Osteoarthritis Who Score Highly on the PainDETECT Questionnaire Present with Multimodality Hyperalgesia, Increased Pain, and Impaired Physical Function. *Clinical Journal of Pain* **2018**, *34*, 15-21, doi:10.1097/AJP.0000000000000504.
33. Rice, D.A.; Kluger, M.T.; McNair, P.J.; Lewis, G.N.; Somogyi, A.A.; Borotkanics, R.; Barratt, D.T.; Walker, M. Persistent postoperative pain after total knee arthroplasty: a prospective cohort study of potential risk factors. *British Journal of Anaesthesia* **2018**, *121*, 804-812, doi:10.1016/j.bja.2018.05.070.
34. Wallis, J.A.; Taylor, N.F.; Bunzli, S.; Shields, N. Experience of living with knee osteoarthritis: A systematic review of qualitative studies. *BMJ Open* **2019**, *9*, 1-11, doi:10.1136/bmjopen-2019-030060.
35. Moreton, B.J.; Tew, V.; Das Nair, R.; Wheeler, M.; Walsh, D.A.; Lincoln, N.B. Pain phenotype in patients with knee osteoarthritis: Classification and measurement properties of painDETECT and self-report leeds assessment of neuropathic symptoms and signs scale in a cross-sectional study. *Arthritis Care and Research* **2015**, *67*, 519-528, doi:10.1002/acr.22431.
36. Kim, S.H.; Yoon, K.B.; Yoon, D.M.; Yoo, J.H.; Ahn, K.R. Influence of Centrally Mediated Symptoms on Postoperative Pain in Osteoarthritis Patients Undergoing Total Knee Arthroplasty: A Prospective Observational Evaluation. *Pain Practice* **2015**, *15*, E46-E53, doi:10.1111/papr.12311.
37. Soni, A.; Wanigasekera, V.; Mezue, M.; Cooper, C.; Javaid, M.K.; Price, Andrew J.; Tracey, I. Central Sensitization in Knee Osteoarthritis: Relating Presurgical Brainstem Neuroimaging and PainDETECT-Based Patient Stratification to Arthroplasty Outcome. *Arthritis and Rheumatology* **2019**, *71*, 550-560, doi:10.1002/art.40749.
38. Nicholls, E.; Thomas, E.; van der Windt, D.A.; Croft, P.R.; Peat, G. Pain trajectory groups in persons with, or at high risk of, knee osteoarthritis: Findings from the Knee Clinical Assessment Study and the Osteoarthritis initiative. *Osteoarthritis and Cartilage* **2014**, *22*, 2041-2050, doi:10.1016/j.joca.2014.09.026.
39. Baron, E.C.; Davies, T.; Lund, C. Validation of the 10-item Centre for Epidemiological Studies Depression Scale (CES-D-10) in Zulu, Xhosa and Afrikaans populations in South Africa. *BMC Psychiatry* **2017**, *17*, 1-14, doi:10.1186/s12888-016-1178-x.
40. Berghmans, D.D.; Lenssen, A.F.; van Rhijn, L.W.; de Bie, R.A. The Patient-Specific Functional Scale: Its Reliability and Responsiveness in Patients Undergoing a Total Knee Arthroplasty. *J Orthop Sports Phys*

Ther **2015**, *45*, 550-556, doi:10.2519/jospt.2015.5825.
41. Stratford, P.; Gill, C.; Westaway, M.; Binkley, J. Assessing disability and change on individual patients: a report of a patient specific measure. *Physiotherapy Canada* **1995**, *47*, 258-263, doi:10.1007/s00586-010-1521-8.
42. Dawson, J.; Fitzpatrick, R.; Murray, D.; Carr, A. Questionnaire on the perceptions of patients about total hip replacement. *The Journal of bone and joint surgery. British volume* **1996**, *80*, 63-69, doi:10.1302/0301-620x.78b5.0780856.
43. Harris, K.; Dawson, J.; Doll, H.; Field, R.E.; Murray, D.W.; Fitzpatrick, R.; Jenkinson, C.; Price, A.J.; Beard, D.J. Can pain and function be distinguished in the Oxford Knee Score in a meaningful way? An exploratory and confirmatory factor analysis. *Quality of Life Research* **2013**, *22*, 2561-2568, doi:10.1007/s11136-013-0393-x.
44. Brady, T.J. Measures of self-efficacy. *Arthritis Care and Research* **2011**, *63*, s473-s473, doi:10.1002/acr.20567.
45. Eyles, J.P.; Hunter, D.J.; Meneses, S.R.F.; Collins, N.J.; Dobson, F.; Lucas, B.R.; Mills, K. Instruments assessing attitudes toward or capability regarding self-management of osteoarthritis: a systematic review of measurement properties. *Osteoarthritis and Cartilage* **2017**, *25*, 1210-1222, doi:10.1016/j.joca.2017.02.802.
46. Peters, M.; Potter, C.M.; Kelly, L.; Fitzpatrick, R. Self-efficacy and health-related quality of life: A cross-sectional study of primary care patients with multi-morbidity. *Health and Quality of Life Outcomes* **2019**, *17*, 1-11, doi:10.1186/s12955-019-1103-3.
47. Lorig, K.; Chastain, R.L.; Ung, E.; Shoor, S.; Holman, H.R. DEVELOPMENT AND EVALUATION OF A SCALE TO MEASURE PERCEIVED SELF-EFFICACY IN PEOPLE WITH ARTHRITIS. *Arthritis and Rheumatism* **1989**, *32*, 37-44.
48. Gates, L.S.; Leyland, K.M.; Sheard, S.; Jackson, K.; Kelly, P.; Callahan, L.F.; Pate, R.; Roos, E.M.; Ainsworth, B.; Cooper, C.; et al. Physical activity and osteoarthritis: a consensus study to harmonise self-reporting methods of physical activity across international cohorts. *Rheumatology International* **2017**, *37*, 469-478, doi:10.1007/s00296-017-3672-y.
49. Fernandez-Fernandez, R.; Rodriguez-Merchan, E.C. Better survival of total knee replacement in patients older than 70 years: a prospective study with 8 to 12 years follow-up. *The archives of bone and joint surgery* **2015**, *3*, 22-28.
50. Wang, X.; Jin, X.; Han, W.; Cao, Y.; Halliday, A.; Blizzard, L.; Pan, F.; Antony, B.; Cicuttini, F.; Jones, G.; et al. Cross-sectional and Longitudinal Associations between Knee Joint Effusion Synovitis and Knee Pain in Older Adults. *Journal of Rheumatology* **2016**, *43*, 121-130, doi:10.3899/jrheum.150355.

-
